# Dose-dependent effects and mechanisms of exercise-like stimulation on cardiac injury and contractile function: outcomes of the MICRO-ATHLETE study

**DOI:** 10.1007/s00395-026-01182-9

**Published:** 2026-04-30

**Authors:** Tom T. J. Luiken, Carla Cofiño-Fabres, José M. Rivera-Arbeláez, Danique Snippert, Ellen J. S. Denessen, Sacha K. Lamers, Nicoleta Cius, Koen van den Dries, Alma M. A. Mingels, Dick H. J. Thijssen, Robert C. J. J. Passier, Thijs M. H. Eijsvogels

**Affiliations:** 1 Department of Medical BioSciences, Radboud University Medical Center, P.O. Box 9101, 6500 HB Nijmegen, The Netherlands; 2 Department of Applied Stem Cell Technologies, TechMed Centre, University of Twente, Enschede, The Netherlands; 3 BIOS Lab-On-a-Chip Group, Max Planck Institute for Complex Fluid Dynamics, MESA+ Institute for Nanotechnology, University of Twente, Enschede, The Netherlands; 4 Department of Clinical Chemistry, Central Diagnostic Laboratory, Maastricht University Medical Center, Maastricht, The Netherlands; 5 CARIM School for Cardiovascular Diseases, Maastricht University Medical Center, Maastricht, The Netherlands; 6 Department of Cardiology, Division of Experimental Cardiology, Erasmus Medical Center, Rotterdam, The Netherlands; 7 Research Institute for Sport and Exercise Sciences, Liverpool John Moores University, Liverpool, UK; 8 Department of Anatomy and Embryology, Leiden University Medical Centre, Leiden, The Netherlands

**Keywords:** Cardiac injury, In vitro exercise, Biomarkers, Cardiac performance, Tissue engineering

## Abstract

Observational studies revealed that exercise produces cardiac troponin release and cardiac fatigue in athletes. However, human in vivo studies offer limited insights into the underlying mechanisms of these potential deleterious effects. This study investigated exercise-induced cardiac troponin release, cardiac fatigue, and its potential mechanisms in a versatile in vitro Engineered Heart Tissue (EHT) model. EHTs were subjected to exercise-like electrical pulse stimulation (EL-EPS) at 2.5 Hz (150 bpm) for 2 or 4 h and compared to non-stimulated controls and doxorubicin (DOX)-exposed tissues. High-sensitive cardiac troponin T (hs-cTnT), lactate dehydrogenase (LDH), and cardiomyocyte contractile function were measured at baseline, immediately after EL-EPS, and after 20 h of recovery. Cell death was quantified by nuclear and mitochondrial DNA concentrations and tissue integrity was assessed using microscopy following exposure. Following 2 and 4 h of EL-EPS, and after DOX exposure, we found increased hs-cTnT and LDH levels and reduced contractile function compared to control. After recovery, contractile function remained impaired after 4 h EL-EPS and DOX exposure, whereas the effects were abolished after recovery for the 2 h EL-EPS group. Elevated nuclear and mitochondrial DNA release was found following DOX, but not after 2 and 4 h EL-EPS. Finally, EL-EPS was associated with the formation of actin aggregates. EL-EPS induced dose-dependent increases in hs-cTnT and LDH concentrations in conjugation with signs of cardiac fatigue, but without elevated markers of permanent cell death (i.e., mitochondrial/nuclear DNA). These findings suggest that exercise-induced elevations of cardiac biomarker concentrations are indicative of reversible cardiac injury.

**Supplementary Information:**

The online version contains supplementary material available at 10.1007/s00395-026-01182-9.

## Introduction

A physically active lifestyle with regular exercise training promotes cardiovascular health and represents an effective strategy to reduce the risk of cardiovascular morbidity and mortality [7]. Exposure to an acute bout of vigorous-intensity exercise, however, produces significant cardiac stress. Previous studies have shown that vigorous exercise, such as long-distance running or cycling, is associated with transient cardiac dysfunction and elevated cardiac troponin (cTn) concentrations in athletes [28, 37, 49]. Elevated cTn concentrations above the assay-specific 99th percentile upper reference limit are indicative of myocardial injury [44] and associated with an increased risk of major adverse cardiovascular events and mortality [5].

Previous studies found that exercise-induced cTn release is primarily influenced by exercise intensity and duration, with greater cTn increases observed after longer and more intense activities [15, 32]. However, due to their observational design, these studies cannot investigate the underlying mechanisms of these cardiac responses. For example, in vivo studies using cardiac imaging techniques cannot distinguish whether exercise-induced cTn release results from necrosis, apoptosis, increased cardiomyocyte turnover, or increased cardiomyocyte permeability [3]. This is relevant as marathon athletes have been reported to exhibit myocardial fibrosis and coronary artery calcification, which impact the magnitude of exercise-induced cTn release and may reflect underlying subclinical myocardial damage [10, 34, 35]. Recent technological advances have enabled the development of three-dimensional (3D) human cardiac in vitro models, that recapitulate key features of the heart, including mechanical contraction, molecular transport, and electrical activity [1, 11]. This offers new opportunities to investigate the acute effects of exercise on cardiomyocytes and, ultimately, to better understand their potentially harmful effects.

The MICRO-ATHLETE study sought to investigate the dose-dependent effects of exercise exposure-like stimulation on cardiac injury and contractile function. For this purpose, we used a 3D Engineered Heart Tissue (EHT) platform in which human induced pluripotent stem cell (hiPSC)-derived cardiomyocytes were subjected to exercise-like electrical pulse stimulation (EL-EPS) [38], mimicking an increased heart rate. We hypothesized that EL-EPS would lead to a dose-dependent increase in biomarkers of cardiac injury (i.e., cTn), accompanied by impaired contractile function of the EHTs. As a secondary exploratory objective, we investigated the effects of EL-EPS on mechanisms of cell death (i.e., the release of nuclear and mitochondrial DNA) and on alterations in tissue integrity.

## Methods

### In vitro engineered heart tissue platform

The MICRO-ATHLETE platform used human-engineered cardiomyocyte tissues to unravel the deleterious effects of **exercise** on the heart. The methodology for creating the EHT platform has been described previously [38]. In short, the platform comprises a commercial 12-well plate equipped with 12 poly-methylmethacrylate (PMMA) holders. Each holder supports one block of polydimethylsiloxane (PDMS) containing six cantilevers that act as an anchor point for three EHTs. Each well contains three molds that were made of a 1.2 mL mixture (1:1) of 2 × cardiomyocyte medium (CM) composed of DMEM (Sigma, St. Louis, MO, USA, D5030), 15 mM glucose, 0.5 mM sodium pyruvate, 0.19 mM sodium hydroxybutyrate, 0.5 mM L-carnitine, 1 mM creatine, 5 mM taurine, phenol red (0.011 g/L), 1X Trace elements (A, B, and C; Corning), 1X chemically defined lipids (Life Technologies, Waltham, MA, USA), 2 mM Glutamax, 400 µM α-thioglycerol, 0.1X ITS-X, 50 µg/mL AA-2P, 0.5% Pen-Strep, 3.5 g/L sodium bicarbonate, 100 nM T3, 100 ng/mL Long R3 IGF-1, and 1 µM dexamethasone [38] and 20% gelatin from porcine skin (Sigma-Aldrich, G1890—1006).

Cardiomyocytes were generated from hiPSC (WTC-11, WT GM25256*G0002), which were differentiated and purified by lactate metabolism selection as previously described [39]. Eight different batches of hiPSC-differentiated cardiomyocytes were used consisting of 91 ± 2% cTnT-positive cells, quantified with flow cytometry. The hiPSC-derived cardiomyocytes and human adult cardiac fibroblasts were resuspended in CM medium, filtered, and counted. EHTs were constructed with a 3% human fibroblast content. Next, an extracellular matrix (ECM) mixture was prepared on ice, comprising 2X CM medium, fibrinogen (2 mg/mL final concentration, Sigma-Aldrich F8630), Matrigel (1 mg/mL final concentration, Sigma Aldrich 354,230), and aprotinin (2.5 µg/mL final concentration, Sigma-Aldrich, A1153). This ECM mixture was then added to the resuspended cells, resulting in a final cell concentration of 16.3 × 10^6 cells/mL. Next, 0.6 U/mL Thrombin (Sigma-Aldrich, T7513) was added to the cells + ECM mixture. Quickly after mixing, 15 µL of the final mix (2.45 × 10^5^ cells) was added to each tissue mold.

Immediately after adding the cells to the gelatin molds, PDMS blocks with three parallel pairs of cantilevers were placed into the cell suspension. These PDMS pillars were fabricated from a mixture of Sylgard 184 curing agent and PDMS (1:10 v/v ratio, Sigma-Aldrich, St. Louis, MO, USA) into a negative Teflon mold [38]. The top of the PDMS pillar was painted with a mixture of PDMS with 13% (*w/w*) black carbon (Vulcan XC 72R) and contained a PDMS disk at the end of the pillar to prevent the tissues from sliding off when upside-down. After 10 min, 1.0 mL of CM culture medium was added to each well. The EHTs were incubated at 37 °C and 10% CO_2_ in a humidified cell culture incubator. The platform accommodates a total of 36 EHTs, distributed across 12 wells. EHTs were incubated for nine days, receiving fresh medium every two days. EHTs that disconnected from their PDMS cantilevers before the start of the experimental study protocol were removed from the platform and excluded from subsequent analysis.

### Study protocol

**Fig. 1:**
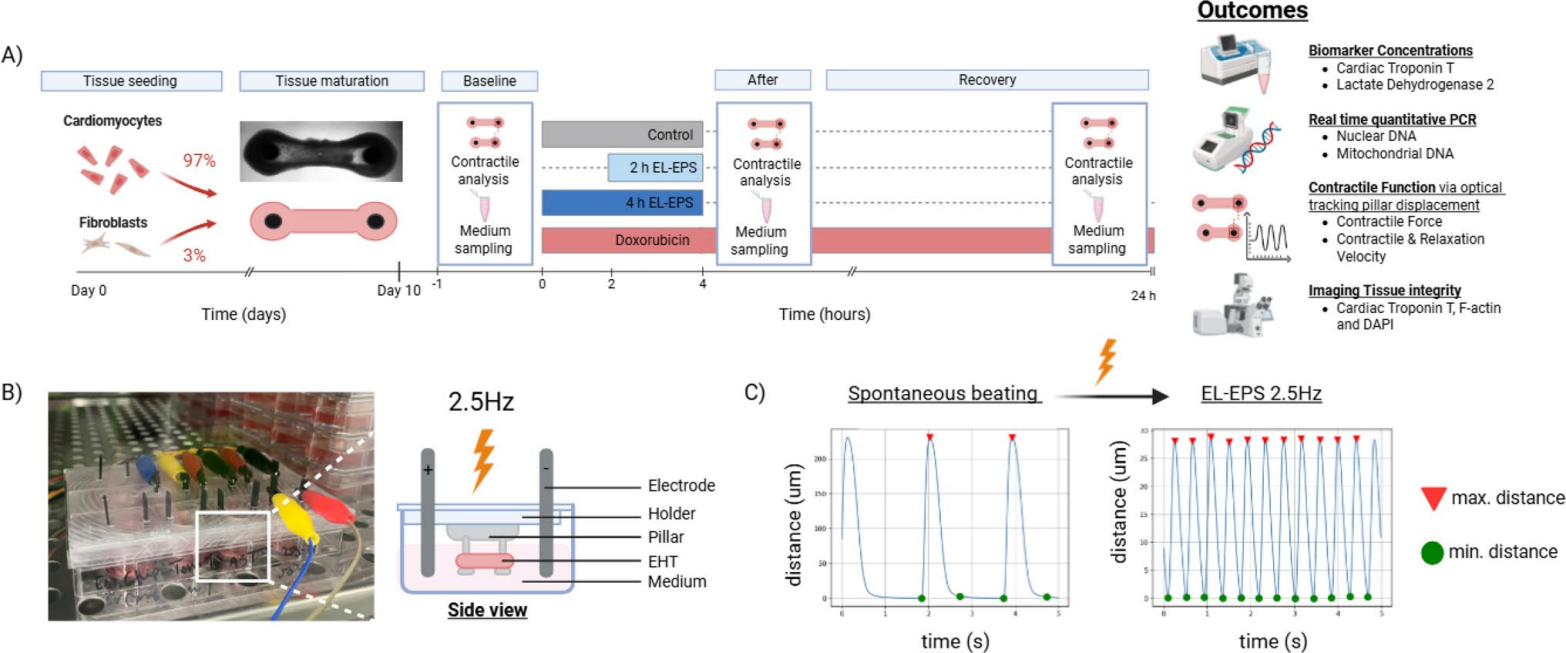
Schematic overview of the MICRO-ATHLETE experimental procedures. **A** EHTs were created using hiPSC-derived cardiomyocytes and cardiac fibroblasts. EHTs were used as non-stimulated controls, electrically stimulated for 2 or 4 h, or treated with Doxorubicin, and analyzed for biomarker concentrations, contractile function, markers of cell death and tissue integrity by imaging. **B** EL-EPS was applied by carbon electrodes in the platform. **C** EL-EPS increased spontaneous beating frequency to 2.5 Hz

EHTs were assigned to i) a control, *ii*) 2 h EL-EPS, *iii*) 4 h EL-EPS, or *iv*) a doxorubicin (DOX)-treated group. At 10 days post-tissue seeding, the medium was sampled and baseline EHTs’ contractile function was measured (Fig. 1A). Subsequently, EHTs were exposed to their respective treatment. EL-EPS was introduced by placing two carbon electrodes (Easycomposites 1 mm Carbon Fiber Rod) into the medium perpendicular to the tissues, approximately 20 mm apart inside the well (Fig. 1B). The carbon electrodes were connected to the pulse generator STG4008 (MultiChannelSystems) and EHTs were electrically stimulated to 2.5 Hz for either 2 or 4 h (10 ms biphasic pulses, 4–5 V/cm) using MC_Stimulus II software (version 3.5.1). Pacing frequency and duration were selected to represent marathon-like conditions, with 2.5 Hz corresponding to 150 beats per minute, a heart rate commonly observed during marathon running [9, 14, 21]. The effect of EL-EPS was compared with a negative, non-stimulated control group and a positive control group treated with 5 µM doxorubicin hydrochloride (Sigma-Aldrich, D1515), a compound well known for its cardiotoxic effects [30, 42]. EHT contractile function was measured [40, 41] and the medium was sampled immediately following exposure. Thereafter, all tissues received a complete CM medium refreshment. DOX treatment was continued for the respective EHTs. After a recovery period of 20 h, a final medium sample was collected and contractile function was measured. EHTs were fixed for subsequent histological assessment.

### Biomarkers of cardiomyocyte injury

Concentrations of high-sensitivity cardiac troponin T (hs-cTnT) were our primary outcome and were measured in the medium at baseline, immediately after exposure, and following 20 h of recovery. At each time point, 0.5 mL of medium was sampled, snap-frozen, and stored at − 80 °C until analysis. hs-cTnT concentrations were measured using the Cobas e402 analyzer with the Elecsys Troponin T Gen 5 assay (Roche Diagnostics), following the manufacturer’s protocol. LDH concentrations were measured using the Cobas c303 analyzer with the Lactate Dehydrogenase 2 assay (Roche Diagnostics) according to the manufacturer’s protocol. Biomarker concentrations were adjusted for the total volume of culture medium and the number of tissues per well.

The presence of mitochondrial DNA (mtDNA) and nuclear DNA (nDNA) was assessed as an indicator of cell death. Quantification of mtDNA and nDNA was performed using primers targeting the human mitochondrial NADH dehydrogenase 1 (*mtND1*) gene and the human β-globin (*HBB*) gene, respectively. DNA was isolated from culture medium collected after 20 h of recovery using the DNeasy Blood and Tissue Kit (QIAGEN, Valencia, CA). Real-Time quantitative PCR (RT-qPCR) was performed in technical replicates using the SensiMix SYBR & Fluorescein kit (Bioline, #QT615—05) on a CFX Opus 384 Real-Time PCR System (Bio-Rad Laboratories). Results were normalized for the culture medium volume and the number of tissues present in each well. Primer sequences are listed in Supplemental Table 1. All primers were obtained from Integrated DNA Technologies (Coralville, IA, USA).

### EHT contractile function

EHT contractile function was assessed at baseline, after exposure, and following 20 h of recovery. Analysis of EHT contractile performance was performed in a Nikon ECLIPSE Ti2 inverted microscope (RRID:SCR_02 1068) under temperature and humidity control (37 °C and 5% CO_2_). EHTs were imaged for 5 s at a frame rate of 100 frames per second and a 2 × magnification (Plan APO 2X/0.10 WD 8.5) using NIS-Elements software (version AR 5.42.07). The contractile function was assessed via optical tracking of the displacement of the black cantilevers in each frame and compared to the known initial distance using Forcetracker software [40]. The main parameter for contractile function, the Force of Contraction (FoC), was calculated using the displacement of the centroid of PDMS pillars according to the elastic beam bending equation as previously established [2]. To further evaluate contractile dynamics, contractile and relaxation velocities were measured to detect early fatigue-related impairments in muscle function and efficiency. The contraction and relaxation velocities were quantified as the displacement amplitude divided by the time to peak contraction and time to peak relaxation, respectively (Fig. 1C). All parameters of contractile function are presented as changes relative to baseline measurements.

### Immunostaining, histology, and imaging

To visualize EHT integrity, whole-mount staining was performed. Tissues were fixed in 4% paraformaldehyde for 45 min, followed by washing with 0.3% Triton X-100 (Sigma-Aldrich; 3 × 20 min). Non-specific binding was blocked overnight at 4 °C using a blocking solution containing 3% BSA (Sigma-Aldrich A9418—100G), 0.3% Triton X-100, and 0.1% Tween 20 in PBS. The primary antibody, anti-cardiac troponin T (1:250; Invitrogen™, MA5—12,960), was applied for 2 days at 4 °C. After incubation, tissues were washed again with 0.3% Triton X-100 (3 × 20 min), followed by incubation with the secondary antibody Donkey anti-Mouse IgG Alexa Fluor 647 (1:500; Invitrogen™, A31571), Phalloidin (Invitrogen™, ActinGreen™ 488 ReadyProbes™ reagent), and DAPI (1:2000, Sigma-Aldrich F6057—20ML) for 24 h at 4 °C. Following three washes with PBS, tissues were mounted onto microscope slides and imaged using a Zeiss LSM 880 confocal microscope (Plan APO 20x/0.8 WD 0.55).

### Statistical analysis

Statistical analysis was conducted using SPSS Statistics Version 29 (IBM Corp., Armonk, NY, USA), and graphs were generated with GraphPad Prism (version 10.2, GraphPad Software, San Diego, CA, USA). Data were visually inspected for normality, checked for kurtosis and skewness, and tested using Shapiro–Wilk tests. Continuous variables were presented as mean with standard deviation (SD) when normally distributed or as median (Q1–Q3) when not normally distributed. Variables were presented as counts and percentages. Natural logarithmic transformation was applied to non-parametric parameters. Baseline group differences and mitochondrial DNA levels were analyzed using One-way ANOVA or the Kruskal–Wallis test for non-parametric parameters. Linear mixed models with restricted maximum likelihood estimation were used to compare differences in EL-EPS-induced changes in biomarker concentrations and contractile function across groups. The model included intervention (control, 2 h EL-EPS, 4 h EL-EPS, and DOX exposure), time points (baseline, after, recovery), and their interaction as fixed effects. Additionally, the correlation between immediate functional adaptations and biomarker release was evaluated using linear regression, relating the average FoC of EHTs within a well to the concentrations of hs-cTnT or LDH. Multiple comparisons were adjusted using Bonferroni post hoc testing. All statistical tests were 2-sided, and *p* values < 0.05 were considered statistically significant.

## Results

We aimed to mimic exercise in vitro via EL-EPS. The durations of 2 and 4 h of EL-EPS were chosen to mimic variation in endurance exercise exposure that is typically observed in Masters’ athletes (i.e., half-marathon vs marathon). A total of 165 EHTs were included in this study, of which 24 were excluded due to technical complications in the electrical stimulation set-up that affected the transmission of the pacing stimulus to the tissues. The remaining 141 EHTs (85%) were randomly assigned to the control (*n* = 45), 2 h EL-EPS (*n* = 42), 4 h EL-EPS (*n* = 41) or DOX (*n* = 13) groups.

### Cardiac injury biomarkers

**Fig. 2:**
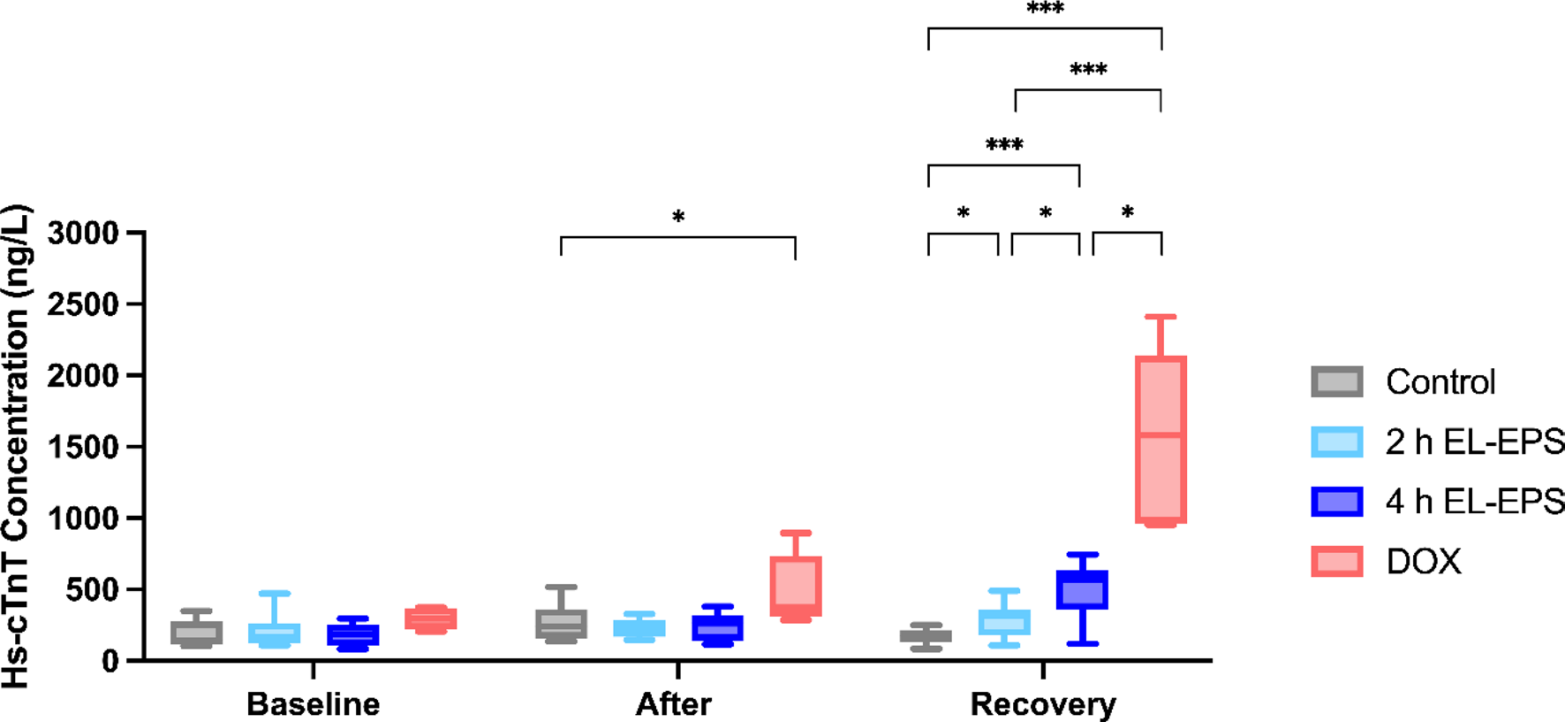
High-sensitivity cardiac troponin T (hs-cTnT) concentrations in non-stimulated controls, after 2 or 4 h of exercise-like electrical pulse stimulation (EL-EPS) or doxorubicin (DOX) treatment. Data are presented as median ± IQR, *p < 0.05, **p < 0.01 and ***p < 0.001

We evaluated the effect of EL-EPS on the release of hs-cTnT, which is widely regarded as a sensitive marker of cardiac injury [44]. Baseline hs-cTnT concentrations (185.4 ng/L [121.6–281.8]) did not differ between groups (*p* = 0.15). Linear mixed model analysis revealed that hs-cTnT concentrations significantly increased across timepoints (*P*_time_ < 0.001), differed between groups (*P*_group_ < 0.001) and showed a significant time*group interaction (*P*_time*group_ < 0.001) (Fig. 2). The DOX group, but not 2 or 4 h EL-EPS groups, showed a higher hs-cTnT concentration compared to the control group immediately following exposure (Supplemental Table 2). After 20 h of recovery, the 2-h and 4 h EL-EPS and DOX groups revealed significantly higher hs-cTnT concentrations compared to the control, with higher hs-cTnT concentrations after 4 h *versus* 2 h of pacing (Fig. 2, Supplemental Table 2).

**Fig. 3:**
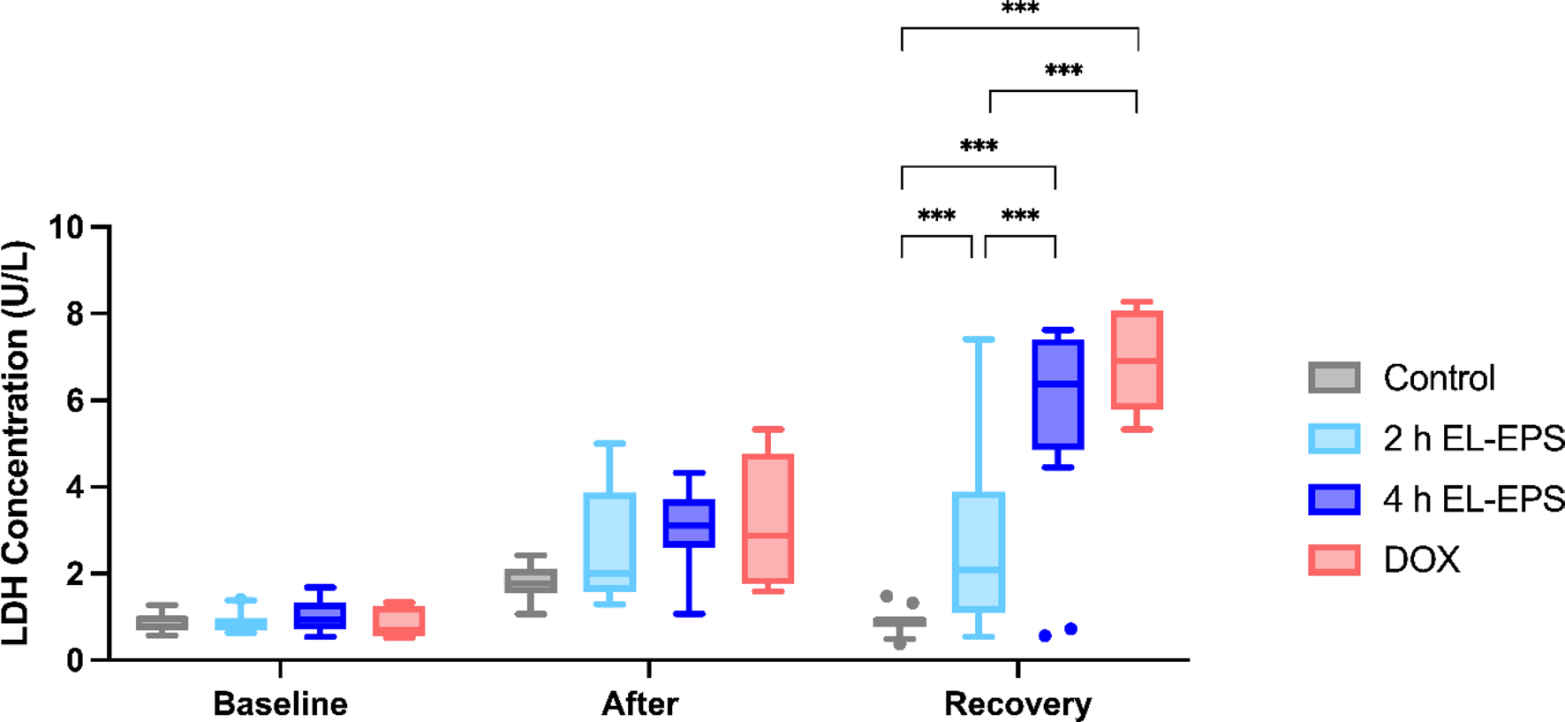
Lactate Dehydrogenase (LDH) concentrations in non-stimulated controls, after 2 or 4 h of exercise-like electrical pulse stimulation (EL-EPS) or doxorubicin (DOX) treatment. Data are presented as median ± IQR, *p < 0.05, **p < 0.01 and ***p < 0.001

Lactate Dehydrogenase (LDH) concentrations were assessed as a measure of cellular injury [6]. Baseline LDH concentrations (0.90 U/L [0.69–1.08]) did not differ between groups (*p* = 0.90). Linear mixed model analysis revealed that LDH increased across time points (*P*_time_ < 0.001), varied between intervention groups (*P*_group_ < 0.001) and showed a significant time*group interaction (*P*_time*group_ < 0.001) (Fig. 3). LDH concentrations did not differ between groups immediately after exposure but were significantly elevated after 20 h of recovery in the 2 and 4 h EL-EPS and DOX groups compared to control (Fig. 3, Supplemental Table 2).

**Fig. 4:**
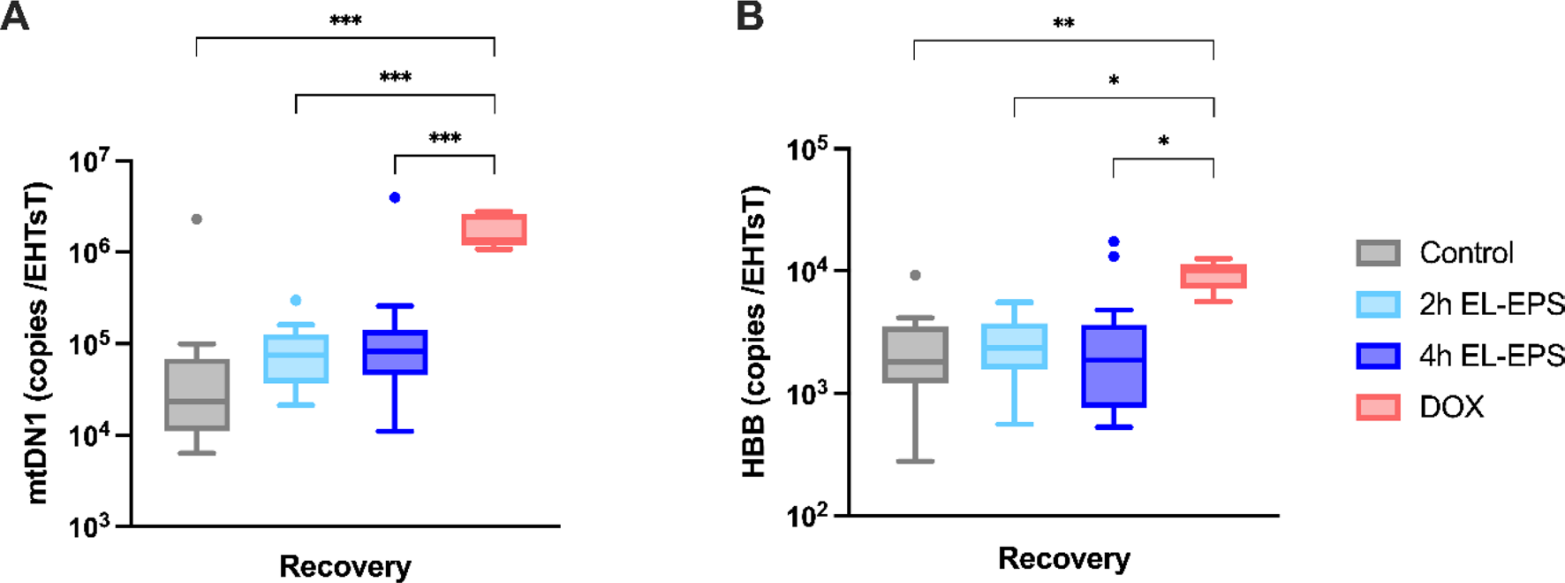
Release of mitochondrial and nuclear DNA at recovery as a marker of cell death. **A** mtDNA copy number and **B** HBB copy number in non-stimulated controls, after 2 or 4 h of exercise-like electrical pulse stimulation (EL-EPS) or doxorubicin (DOX) treatment, measured following 20 h of recovery. Data are presented as median ± IQR. Statistical significance: *p < 0.05, **p < 0.01, ***p < 0.001

To determine whether EL-EPS caused actual cell death, we measured nuclear and mitochondrial DNA release, as definitive markers of cell death. After 20 h of recovery, the mtND1 copy number in the DOX group (*p* < 0.001), but not in the 2 and 4 h EL-EPS groups (*p* = 0.83 and *p* = 0.23, respectively), was significantly higher compared to the control group (Fig. 4A, Supplemental Table 2). Similarly, HBB copy number was significantly higher in the DOX versus control group (*p* = 0.006), whereas the 2 and 4 h EL-EPS groups (*p* = 1.00 and *p* = 1.000, respectively) did not differ from control (Fig. 4B, Supplemental Table 2). Seven samples (*n* = 3 for Control, *n* = 1 for 2 h EL-EPS, and *n* = 3 for 4 h EL-EPS) had high Cq variation due to low HBB copy number, but exclusion of these samples did not alter our findings (Supplemental Fig. 1), suggesting that EL-EPS did not induce measurable cell death under these conditions.

### EHT contractile function

**Fig. 5:**
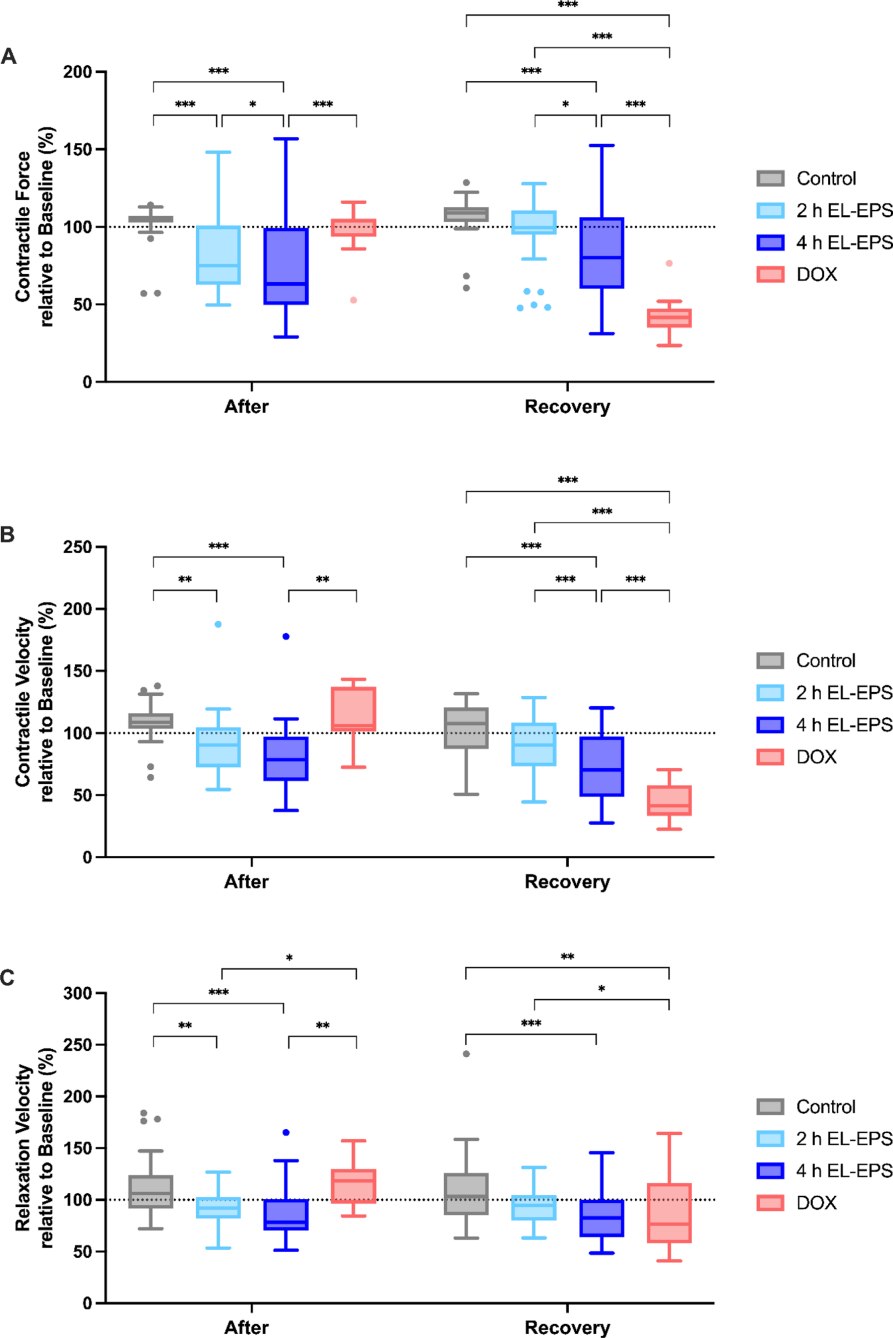
Changes in contractile function in non-stimulated controls, after 2 or 4 h of exercise-like electrical pulse stimulation (EL-EPS) or doxorubicin (DOX) treatment. Alterations in contractile function relative to baseline were assessed by **A** Force of Contraction, **B** Contractile Velocity, and **C** Relaxation Velocity. Data are presented as median ± IQR, *p < 0.05, **p < 0.01 and ***p < 0.001

**Fig. 6:**
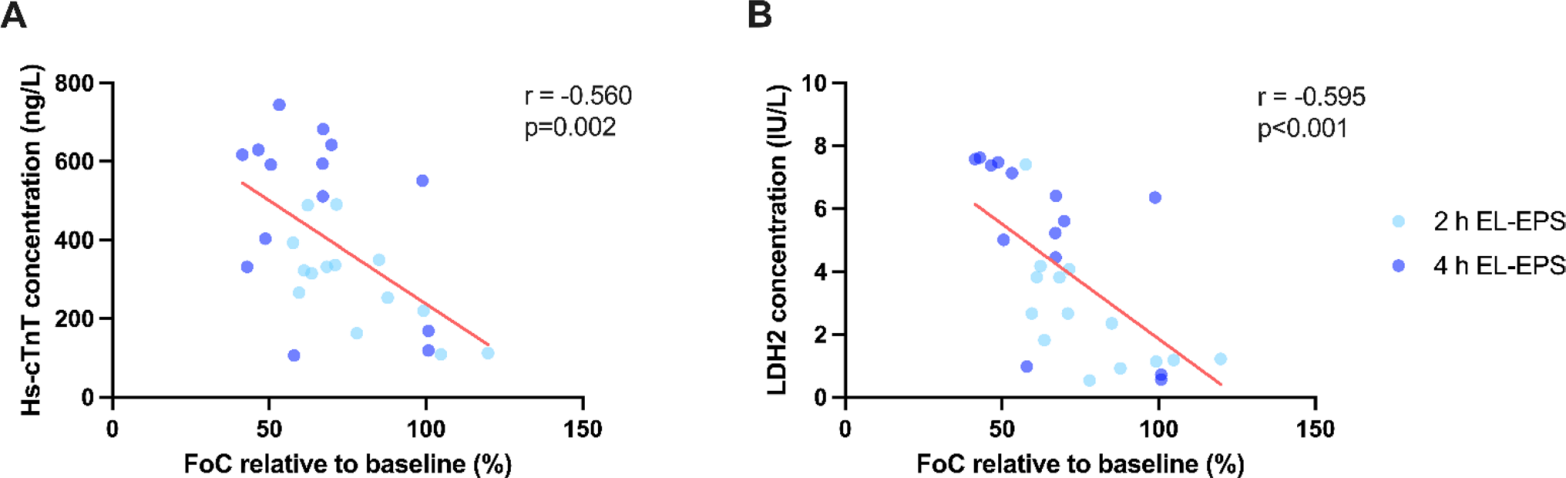
Correlation of relative changes in force of contraction (FoC) following exposure with **(A)** hs-cTnT and **(B)** LDH concentrations following recovery

Contractile function of EHTs was assessed to determine whether EL-EPS alters functional performance. Baseline FoC (290 µN [258–331]) did not differ between groups (*p* = 0.13). Linear mixed model analysis revealed that FoC concentrations significantly increased across time points (*P*_time_ < 0.001), differed between groups (*P*_group_ < 0.001) and showed a significant time*group interaction (*P*_time*group_ < 0.001) (Fig. 5A). We observed a significantly lower FoC immediately following 2 and 4 h EL-EPS exposure, in a dose-dependent manner, compared to control. After 20 h of recovery, FoC remained significantly lower in the 4 h EL-EPS and DOX group compared to control, whereas the immediate reduction in FoC observed in the 2 h EL-EPS group was attenuated (Fig. 5A, Supplemental Table 2). A significant inverse correlation was found between the relative changes in FoC following EL-EPS and hs-cTnT (*r* = -0.560, *p* = 0.002) and LDH concentrations (*r* = -0.595, *p* = 0.004, Fig. 6).

At baseline, contractile velocity (1340 µm/s [1135–1681]) did not differ between groups (*p* = 0.05), whereas relaxation velocity differed between groups (*p* = 0.037). Specifically, relaxation velocity was slightly but significantly lower in DOX vs 2 h EL-EPS (292 µm/s [247–385] versus 376 µm/s [323–422], *p* = 0.04), but not compared to control (347 µm/s [312–403]) or 4 h EL-EPS (337 µm/s [305–402]). Linear mixed model analysis revealed significantly increased contractile and relaxation velocities across timepoints (P_time_ < 0.001), with differences between groups (*P*_group_ < 0.001) and a significant time*group interaction (*P*_time*group_ < 0.001) (Fig. 5B–C). We found significantly lower contractile and relaxation velocities following 2 and 4 h EL-EPS exposure, in a dose-dependent manner, compared to control. After 20 h of recovery, contractile and relaxation velocities were significantly lower in the 4 h EL-EPS and DOX groups compared to control, whereas no change in contractile velocity was found at 20 h recovery for the 2 h EL-EPS group (Fig. 5B–C, Supplemental Table 2).

### Histopathology

**Fig. 7:**
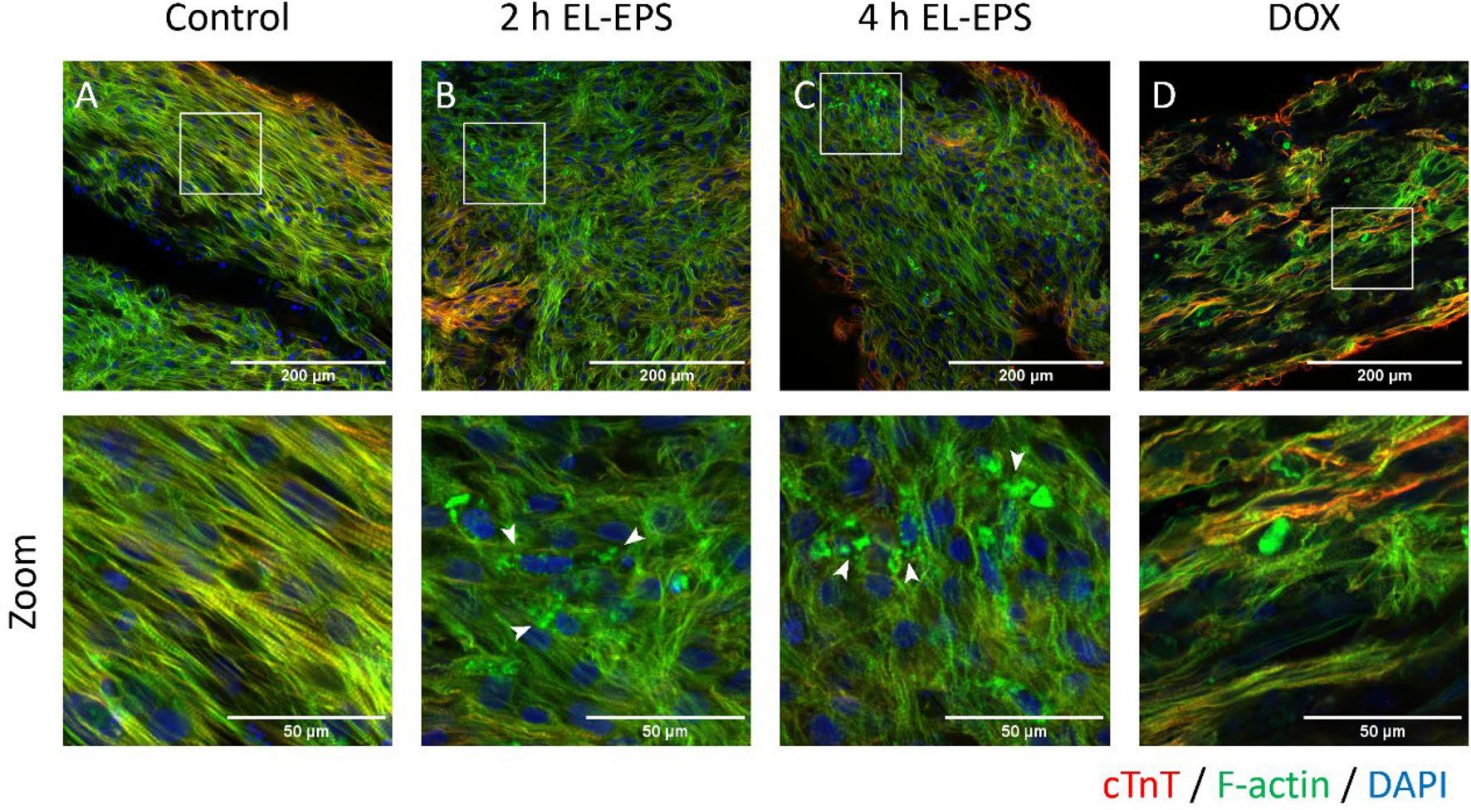
Morphological analysis of non-stimulated controls, 2 and 4 h EL-EPS or doxorubicin (DOX)-exposed EHTs. Representative whole-mount tissue immunostaining of **A** control; **B** 2 h EL-EPS; **C** 4 h EL-EPS; and **D** DOX-exposed EHTs following 20 h of recovery. Sections were immunostained for cardiac troponin T (cTnT, red), Phalloidin (F-actin, green), and DAPI (nuclei, blue). White arrowheads depict the presence of actin aggregates

Changes in EHT tissue integrity following EL-EPS were analyzed in fixed tissues after 20 h of recovery by labeling with cardiac troponin T, Phalloidin, and DAPI (Fig. 7). Immunohistochemical analysis revealed that cardiomyocytes of the unstimulated control group and 2 and 4 h EL-EPS tissues were nicely aligned to the longitudinal axis of the tissues and contained better-organized striated sarcomeres compared to Doxorubicin-exposed EHT. Actin aggregates were observed following 2 and 4 h EL-EPS (Fig. 7B, C indicated by the white arrows), whereas this was not observed in the control and DOX-exposed EHTS, suggesting that EL-EPS may cause structural tissue remodeling.

## Discussion

This study aimed to investigate exercise-induced cTn release, alterations in cardiac function, and its potential mechanisms via electrical stimulation of EHTs. We found that EL-EPS produced a dose-dependent increase in hs-cTnT and LDH concentrations, indicative of cardiomyocyte injury. These changes were accompanied by dose-dependent reductions in contractile function following 2 and 4 h of EL-EPS, which recovered after 20 h following 2 but not 4 h of EL-EPS, indicating transient contractile dysfunction. Despite these changes, neither 2 nor 4 h of EL-EPS caused the release of mitochondrial and nuclear DNA, suggesting that EL-EPS did not induce permanent cell death. Moreover, EL-EPS was associated with the formation of actin aggregates, which might be indicative of structural remodeling. Collectively, these findings demonstrate that EL-EPS induces dose-dependent biomarker release and transient contractile dysfunction without evidence of permanent cell death, suggesting that exercise-induced cTn release likely reflects a physiological response rather than permanent injury.

Previous in vivo studies have shown that athletes engaged in endurance exercise show transient elevations of (hs-)cTnT concentrations, whereas the magnitude of this release is positively related to the duration and intensity of the exercise exposure [15, 32, 49]. Consistent with these in vivo observations*,* and confirming our hypothesis, we found increases of hs-cTnT concentrations following in vitro EL-EPS of EHTs, with greater elevations following 4 *versus* 2 h of EL-EPS. The magnitude of these responses (2.9- and 1.7-fold increases for 4 and 2 h of EL-EPS, respectively) aligned with those observed in endurance athletes [26]. While in vivo studies typically show elevations immediately following exercise, we observed cTnT elevations at the recovery phase. These distinct release patterns may be attributable to the absence of active tissue perfusion in our EHT model, delaying the release of cTnT into the culture medium opposed to direct increases in circulating cTnT observed in athlete studies [8, 19, 25]. Taken together, our novel and versatile EHT platform successfully reproduced exercise-induced cTnT release in a controlled in vitro setting.

The dose-dependent increases in LDH concentrations further confirmed the presence of exercise-induced cellular injury. LDH elevations are often interpreted as a marker of permanent cell damage, but may also result from physiological processes such as increased lactate metabolism, an increased cellular turnover rate [17] or cardiomyocyte bleb formation [22]. LDH release could, therefore, result from sublethal membrane damage, similar to elevated cTnT concentrations following exercise-induced compromise of cardiomyocyte integrity [4, 47]. Therefore, we additionally assessed the release of mitochondrial and nuclear DNA as definitive markers of cell death. EL-EPS did not increase mtDN1 or HBB copy numbers compared with controls, indicating that despite LDH elevations, permanent cell death may not have occurred. Supporting this reasoning, our positive control demonstrated that DOX treatment consistently induced cell death based on elevated LDH, mtDN1 and HBB copies, aligning with observations from prior reports [36]. Our findings expand on recent observations that an increased beating rate of hiPSC-derived cardiomyocytes and zebrafish hearts was associated with the release of cTnI, but without the presence of cell death [22]. Collectively, these data indicate that the release of hs-cTnT and LDH following EL-EPS likely reflects reversible cardiac damage rather than permanent cell death.

EL-EPS led to an immediate reduction in contractile function, evidenced by decreased contractile force as well as delayed contraction and relaxation velocities, with these effects being dependent on pacing duration. These reductions in contractile function may reflect cardiac fatigue, a common phenomenon which is characterized by a temporal exercise-dose-dependent functional decline in contractile properties [20, 27, 46]. Functional properties following 4 h of EL-EPS did not fully recover after 20 h of recovery and may reflect myocardial stunning, a condition of prolonged yet reversible contractile dysfunction [24]. Myocardial stunning has previously been reported following strenuous cardiac stress in marathon athletes, where exercise-induced cardiac dysfunction may take 1–4 weeks to recover after cessation of exercise [28, 37]. Additionally, we found that the dose-dependent reductions in FoC following 2 and 4 h EL-EPS were inversely related to hs-cTnT concentrations at recovery, which is in line with a meta-analysis that found that post-exercise cTn concentrations were correlated with reductions in diastolic function [13]. The joint observations across in vivo and in vitro studies highlight the presence of an inverse relationship between markers of cardiac injury and temporal cardiac dysfunction [28, 37, 49].

Actin aggregates were found following exposure to EL-EPS but not to DOX. These aggregates may arise from mechanical stretching, as this stimulus enhances actin synthesis and remodeling [48], ultimately contributing to muscle stabilization [29]. Alternatively, actin aggregation may also indicate permanent cytoskeletal damage within muscle fibers, particularly following intense or unaccustomed exercise [33], leading to promotion of fibroblast proliferation, excessive extracellular matrix deposition and fibrosis [43]. We previously hypothesized that long-term exposure to vigorous endurance exercises may link cardiac biomarker release to myocardial fibrosis [18, 45]. However, 2 h EL-EPS induced alterations in contractile function were reversible, and mtDNA and nDNA remained similar to unstimulated tissues. Therefore, the formation of actin aggregates following EL-EPS may represent a physiological rather than pathological response. Future studies are warranted to investigate how EL-EPS may trigger such muscular adaptations.

### Limitations

To our knowledge, this is the first study to mimic exercise within an in vitro platform to better understand the potential deleterious effects of exercise on the human heart. However, some limitations should be considered. First, our platform aims to investigate the effects of vigorous endurance exercise on the heart, but it does not replicate the complex, multi-organ interactions involved in exercise. Since physiological responses to exercise rely on coordinated activity across systems like the cardiovascular, respiratory, and endocrine systems, isolated tissue models cannot fully capture the mechanisms driving exercise-induced cardiac adaptations [23]. Nonetheless, current insights into biomarker release and its underlying mechanisms remain poorly understood because of these multi-organ effects, and dedicated approaches, such as our MICRO-ATHLETE study, are needed to examine isolated pathways. Future studies that incorporate additional physiological components, such as catecholamine release and cytokine signaling, would further enhance our understanding of exercise-induced cardiac responses. Moreover, exercise-induced cardiac troponin release may be influenced by the cardiovascular health status and presence of markers of atherosclerosis and fibrosis [10, 34, 35], which could not be replicated within our current model that uses a priori healthy hi-PSC-derived cardiomyocytes. While cTn elevations are often considered a physiological response, in certain subgroups they may reflect underlying cardiovascular pathology rather than purely reversible cardiomyocyte stress [31]. Second, hiPSC-derived cardiomyocytes do not generally exhibit a fully mature phenotype in terms of structure, contractility, electrophysiology, and gene expression, which remains a major challenge in tissue engineering [16]. Nevertheless, the platform recapitulated exercise-induced cardiac biomarker release that is also observed in (adolescent) athletes [2–4], suggesting that the maturity status of the EHTs does not impact our findings. Finally, we solely investigated the effect of a single-frequency, short-duration electrical stimulation protocol. This was primarily dictated by the maturation state of the EHTs, which exhibited reduced adherence under higher-frequency pacing conditions (> 2.5 Hz). Future studies may elaborate on these findings by examining the impact of varying pacing frequencies and durations on tissue function and biomarker release by employing alternative platforms, such as the micro-EHT model, which is better suited to sustain higher-frequency stimulation [12].

## Conclusion

MICRO-ATHLETE mimicked the effects of endurance exercise through EL-EPS of EHTs in a novel and versatile human cardiac in vitro model. We found that EL-EPS: (i) induces a dose-dependent increase in hs-cTnT and LDH concentrations, indicative of cellular injury; (ii) leads to dose-dependent reductions in contractile function after 2 and 4 h of EL-EPS, which only recovered for the 2 h EL-EPS following 20 h of recovery; and (iii) does not trigger the release of mitochondrial or nuclear DNA, suggesting that EL-EPS does not induce permanent cell death. Collectively, these findings suggest that exercise-induced elevations of cardiac biomarker concentrations are indicative of reversible cardiac injury rather than permanent cell death.

## Supplementary Information

Below is the link to the electronic supplementary material.

Supplementary file1 (DOCX 116 KB)

## Data Availability

The data that support the findings of this study are available from the corresponding author upon reasonable request.

## Abbreviations

(hs-)cTnT: (High-sensitivity) cardiac troponin T
3D: Three-dimensional
EHT: Engineered Heart Tissue
hiPSC: Human-induced pluripotent stem cell
EL-EPS: Exercise-like electrical pulse stimulation
PMMA: Poly-methylmethacrylate
PDMS: Polydimethylsiloxane
CM: Cardiomyocyte medium
ECM: Extracellular matrix
DOC: Doxorubicin
LDH: Lactate dehydrogenase
mtDNA: Mitochondrial DNA
nDNA: Nuclear DNA
*mtND1*: NADH dehydrogenase 1
HBB: Human β-globin
RT-qPCR: Real-Time quantitative PCR
FoC: Force of Contraction
