# Supplementary material for: Dose-dependent effects and mechanisms of exercise-like stimulation on cardiac injury and contractile function: outcomes of the MICRO-ATHLETE study
Source: Basic Res Cardiol. 2026 Apr 30;121(4):725–37. doi: 10.1007/s00395-026-01182-9 (PMC13373019; doi:10.1007/s00395-026-01182-9)
Supplement: Supplementary file 1 — Supplementary file1 (DOCX 116 KB) [file 395_2026_1182_MOESM1_ESM.docx]

SUPPLEMENTAL MATERIAL TO:

**Dose-dependent effects and mechanisms of exercise-like stimulation on cardiac injury and contractile function:
outcomes of the MICRO-ATHLETE study**

**SHORT TITLE**: *In vitro* exercise and troponin release

**Journal**: Basic Research in Cardiology

Tom T.J. Luiken, MSc^a^

Carla Cofiño-Fabres, PhD^b^

José M. Rivera-Arbeláez, PhD^b,c^

Danique Snippert, MSc^b^

Ellen J.S. Denessen, PhD^d,e^

Sacha K. Lamers, MSc^d,e^

Nicoleta Cius, MSc^a,f^

Koen van den Dries, PhD^a^

Alma M.A. Mingels, PhD^d,e^

Dick H.J. Thijssen, PhD^a,g^

Robert C.J.J. Passier, PhD^b,h^

Thijs M.H. Eijsvogels, PhD^a^

1. Department of Medical BioSciences, Radboud University Medical Center, Nijmegen, The Netherlands
2. Department of Applied Stem Cell Technologies, TechMed Centre, University of Twente, Enschede, The Netherlands
3. BIOS Lab-on-a-Chip Group, MESA+ Institute for Nanotechnology, Max Planck Institute for Complex Fluid Dynamics, University of Twente,
4. Department of Clinical Chemistry, Central Diagnostic Laboratory, Maastricht University Medical Center, Maastricht, The Netherlands
5. CARIM School for Cardiovascular Diseases, Maastricht University Medical Center, Maastricht, The Netherlands
6. Department of Cardiology, Division of Experimental Cardiology, Erasmus Medical Center, Rotterdam, The Netherlands
7. Research Institute for Sport and Exercise Sciences, Liverpool John Moores University, United Kingdom
8. Department of Anatomy and Embryology, Leiden University Medical Centre, Leiden, The Netherlands

**Keywords:** Cardiac injury, *In vitro* exercise, Biomarkers, Cardiac performance, Tissue engineering

**Address of correspondence:**

Dr Thijs Eijsvogels, Department of Medical BioSciences (928), Radboud University Medical Center, P.O. Box 9101, 6500 HB Nijmegen, The Netherlands, Tel +31 24 36 13 674, E-mail: Thijs.Eijsvogels@Radboudumc.nl


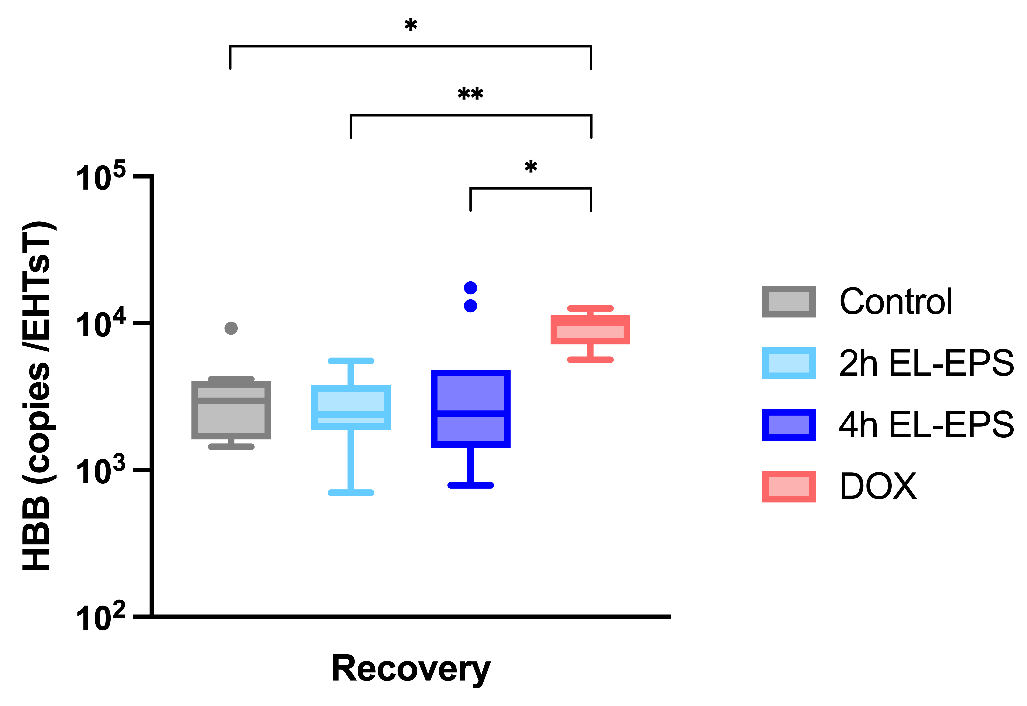


***Supplemental Figure 1.*** *HBB copy number following 2 and 4 h of exercise-like electrical pulse stimulation (EL-EPS) or doxorubicin treatment at 20 hours of recovery. Data point with high Cq variation with sample measurements were removed from analysis. Data are presented as median ± IQR. Statistical significance: *p < 0.05, **p < 0.01, ***p < 0.001.*

| **Supplemental Table 1. Primers for RT-qPCR** | | |
| --- | --- | --- |
| **Primer** | **Forward (5’-3’)** | **Reverse (3’-5’)** |
| Human *mtND1* | ATACCCATGGCCAACCTCCT | GGGCCTTTGCGTAGTTGTAT |
| Human b-globin (*HBB*) | GTGCATCTGACTCCTGAGGAGA | CCTTGATACCAACCTGCCCAG |

| **Supplemental Table 2.** | | | | | |
| --- | --- | --- | --- | --- | --- |
|  |  | Control | 2h EL-EPS | 4h EL-EPS | Doxorubicin |
| Hs-cTnT (ug/L) | |  |  |  |  |
|  | Baseline | 142.5 [117.2-276.0] | 164.5 [120.2-264.0] | 186.8 [107.3-256.3] | 296.0 [221.3-369.0] |
|  | After | 242.3 [156.9-362.2] | 215.3 [168.2-284.7] | 262.7 [141.6-319.8] | **374.4 [309.9-734.6] *** |
|  | Recovery | 177.2 [133.6-215.9] | **319.3 [179.4-360.8] *** | **571.3 [358.5-632.9] *** | **1582.4 [963.4-2137.7] *** |
| LDH (U/L) | |  |  |  |  |
|  | Baseline | 0.95 [0.70-1.03] | 0.86 [0.69-0.97] | 0.94 [0.70-1.33] | 0.69 [0.55-1.26] |
|  | After | 1.77 [1.54-2.11] | 2.00 [1.58-3.87] | 3.11 [2.60-4.19] | 2.88 [1.76-4.78] |
|  | Recovery | 0.93 [0.77-0.98] | **2.09 [1.10-3.89] *** | **6.38 [4.86-7.41] *** | **6.90 [5.89-8.08] *** |
| mtDN1 (copy number) | |  |  |  |  |
|  | Recovery | 27174  [12614-68936] | 75047  [36935-127822] | 82502  [45780-142633] | **1355969**  **[1195854-2593426] *** |
| HBB (copy number) | |  |  |  |  |
|  | Recovery (all) | 1819 [1218-3518] | 2354 [1579-3744] | 1879 [759-3662] | 10123 [7185-11398] * |
| Force of Contraction | |  |  |  |  |
|  | Baseline (μN) | 284 [256-324] | 304 [267-333] | 299 [265-346] | 254 [229-291] |
|  | After (% of baseline) | 104.8 [103.0-107.3] | **75.0 [62.5-100.9] *** | **63.2 [49.7-99.5] *** | 104.0 [93.7-105.2] |
|  | Recovery (% of baseline) | 108.8 [103.2-112.8] | 99.6 [95.0-110.7] | **80.2 [60.1-106.2] *** | 41.7 [35.0-47.4] |
| Contractile velocity | |  |  |  |  |
|  | Baseline (μm/s) | 1331 [1103-1632] | 1418 [1200-1722] | 1329 [1134-1779] | 1164 [961-1645] |
|  | After (% of baseline) | 108.8 [103.5-115.9] | **90.3 [72.6-104.7]*** | **78.8 [61.5-97.1] *** | 106.0 [101.4-137.3] |
|  | Recovery (% of baseline) | 107.9 [87.3-120.7] | 90.4 [73.3-108.4] | **70.3 [48.8-97.4] *** | 41.6 [33.4-57.9] |
| Relaxation velocity | |  |  |  |  |
|  | Baseline (μm/s) | 347 [312-403] | 376 [323-422] | 337 [305-402] | 293 [247-385] |
|  | After (% of baseline) | 106.1 [91.4-123.7] | **92.2 [82.1-102.9] *** | **78.1 [70.4-100.8] *** | 118.2 [96.4-129.8] |
|  | Recovery (% of baseline) | 103.4 [85.3-126.2] | 94.8 [80.0-104.6] | **82.7 [64.2-100.1] *** | 76.4 [58.1-116.3] |
|  | | | | | |
| Values are presented as median [Q1-Q3], n (%), or mean ± SD. *****p < 0.05 vs. Control  Hs-cTnT = High-sensitivity cardiac troponin T; LDH = Lactate dehydrogenase; mtDN1 = NADH dehydrogenase 1; HBB = Human β-globin | | | | | |
